# Lung fibrosis in sarcoidosis. Is there a place for antifibrotics?

**DOI:** 10.3389/fphar.2024.1445923

**Published:** 2024-08-30

**Authors:** Karol Bączek, Wojciech Jerzy Piotrowski

**Affiliations:** Department of Pneumology, Medical University of Łódź, Łódź, Poland

**Keywords:** sarcoidosis, fibrosis, antifibrotic agent, pirfenidone, nintedanib, granuloma

## Abstract

Sarcoidosis, an enigmatic disease with unknown etiology, is characterized by inflammation and the potential involvement of various organs, predominantly the lungs and intrathoracic lymph nodes. Non-caseating granulomas can resolve spontaneously in approximately 60% of cases within 2–3 years. However, sarcoidosis-related mortality has increased. Lung fibrosis, affecting up to 20% of sarcoidosis patients, stands out as a primary cause of mortality. Traditionally, fibrosis is viewed because of prolonged inflammation, necessitating anti-inflammatory treatment with systemic steroids, immunosuppressants, and anti-TNF agents to manage the disease. The recent introduction of antifibrotic drugs such as nintedanib and pirfenidone offers new avenues for treating fibrotic sarcoidosis. Nintedanib, effective in idiopathic pulmonary fibrosis (IPF) and systemic sclerosis-related interstitial lung disease (SSc-ILD), has shown promise in patients with various progressive fibrosing interstitial lung diseases (PF-ILD), including those with sarcoidosis. Pirfenidone, also effective in IPF, has demonstrated potential in managing fibrotic sarcoidosis, though results have been inconclusive due to limited participant numbers in studies. This review explores the theoretical and empirical evidence supporting the use of antifibrotics in sarcoidosis, weighing the benefits and drawbacks. While antifibrotics offer a potential therapeutic approach, further randomized controlled trials are essential to determine their efficacy in fibrotic sarcoidosis. Addressing fibrosis as a continuum of chronic inflammation, the role of antifibrotics in managing sarcoidosis remains an area requiring more in-depth research to improve patient outcomes and advance treatment paradigms.

## Highlights

•  Disease Overview: Sarcoidosis is characterized by inflammation and granuloma formation in various organs, predominantly the lungs and intrathoracic lymph nodes, with a generally favorable prognosis as granulomas resolve spontaneously in 60% of cases within 2–3 years.
•  Mortality and Risk Factors: Recent data show increased sarcoidosis-related mortality, especially among non-Hispanic black females aged 55 and older, challenging the previous perception of the disease as benign. Lung fibrosis, affecting up to 20% of patients, is a major cause of mortality, necessitating timely recognition and management.
•  Traditional Treatments: Anti-inflammatory drugs, including systemic steroids, immunosuppressants, and anti-TNF agents, are the cornerstone of sarcoidosis treatment, targeting inflammation to prevent fibrosis. Treatment is indicated for significant symptomatic disease, progressive lung changes, critical organ involvement, and severe manifestations.
•  Emergence of Antifibrotics: Nintedanib and pirfenidone, effective in other fibrotic lung diseases, offer new avenues for treating fibrotic sarcoidosis. Nintedanib has shown promise in progressive fibrosing ILDs, including sarcoidosis, but more randomized controlled trials (RCTs) are needed to confirm its efficacy.
•  Pathogenesis and Future Research: Sarcoidosis shares pathophysiological characteristics with idiopathic pulmonary fibrosis (IPF) and other fibrotic diseases. The role of antifibrotics in sarcoidosis, particularly their impact on granuloma formation and inflammation, requires further investigation through RCTs to improve patient outcomes and advance treatment paradigms.

## Introduction

Sarcoidosis is an enigmatic disease with an unknown etiology, characterized by inflammation and the potential involvement of various organs within the body. In more than 90% of patients, non-caseating granulomas develop in intrathoracic lymph nodes and lungs. Fortunately, the overall prognosis is favorable; spontaneous healing of granulomas occurs in approximately 60% of affected individuals within 2–3 years. This often results in the complete resolution of lung radiological changes or the presence of only minor residual lesions. Deaths attributable to sarcoidosis are rare, and historically, the condition has been perceived as not limiting lifespan. However, recent data from the United States of America reveal a significant increase in sarcoidosis-related mortality rates between 1988 and 2007. This trend is particularly pronounced among non-Hispanic black females aged 55 years or older. This shift challenges previous perceptions of the disease as benign, prompting a need for further research and understanding of the factors contributing to this observed increase in mortality rates (Swigris et al., 2011). Specific risk factors included lung fibrosis with pulmonary hypertension and cardiac sarcoidosis (Casipit et al., 2023). Similar trends have been reported from other regions of the world (Harada et al., 2022; Ma et al., 2022).

Extensive lung fibrosis stands out as a primary cause of mortality in sarcoidosis, affecting up to 20% of individuals with lung involvement. This severe fibrotic transformation of lung tissue poses a significant threat to the overall health and prognosis of patients with sarcoidosis. Timely recognition and management of such cases are essential for optimizing patient care and outcomes (Patterson and Strek, 2013). Following a traditional perspective, fibrosis is viewed as a continuum or a consequence of a prolonged inflammatory process. This process results in an exaggerated and uncontrolled collagen deposition, gradually replacing granulomas. This understanding underscores the importance of addressing and managing the underlying inflammatory mechanisms to potentially mitigate or prevent the development of extensive fibrosis in individuals with sarcoidosis.

Anti-inflammatory drugs constitute the cornerstone of sarcoidosis treatment. The three primary lines of therapy—systemic steroids, conventional immunosuppressive drugs, and anti-TNF agents—are integral in reducing inflammation, limiting granuloma volume, and preventing fibrosis development. These therapeutic approaches aim to modulate the immune response and manage the inflammatory cascade, ultimately improving clinical outcomes for individuals with sarcoidosis (Baughman et al., 2021a). Indications for treatment are primarily reserved for cases characterized by significant symptomatic disease, causing a notable impairment in the quality of life. Treatment is also warranted in situations involving considerable progression of lung changes, critical internal organ involvement (such as cardiac sarcoidosis and neurosarcoidosis), severe eye manifestations, and the presence of extensive and cosmetically significant skin lesions. These criteria help guide healthcare professionals in determining therapeutic interventions, ensuring that treatment is targeted toward managing the most impactful aspects of sarcoidosis on an individual’s health and wellbeing (Baughman et al., 2021a). The patient’s perspective should always be given due consideration in the decision-making process. This approach aligns with the principles of shared decision-making, where healthcare providers work collaboratively with patients to make decisions informed by medical expertise and the individual’s unique circumstances and preferences.

A recent addition to the spectrum of treatment options is the emergence of nintedanib, an antifibrotic drug, following the publication of results from the INBUILD study. This study substantiates the efficacy of nintedanib in patients diagnosed with progressively fibrosing interstitial lung disease (PF-ILD) (Flaherty et al., 2019). Indeed, nintedanib has been previously employed in the treatment of idiopathic pulmonary fibrosis (IPF), demonstrating its efficacy in slowing disease progression. Notably, it has more recently gained approval for the treatment of systemic sclerosis-related interstitial lung disease (SSc-ILD). This approval is grounded in the positive outcomes observed in several well-designed randomized clinical trials, further expanding the therapeutic applications of nintedanib in the realm of interstitial lung diseases. Such advancements signify the ongoing efforts to identify effective treatments for a broader spectrum of fibrotic lung conditions, contributing to improved patient care and outcomes (Distler et al., 2019; Richeldi et al., 2014). The INBUILD study encompassed a diverse population of patients experiencing a range of progressively fibrosing interstitial lung diseases (ILD). This included conditions such as hypersensitivity pneumonitis (HP), autoimmune-related ILD, unclassified ILD, and pneumoconiosis. Notably, individuals with sarcoidosis who met the criteria for the progressive fibrosing phenotype were also included in the study. It is worth mentioning, however, that the number of participants with sarcoidosis in the study was limited. This diversity in the patient population helps broaden our understanding of the potential applications of nintedanib in various fibrotic ILDs, although the specific impact on sarcoidosis may require further investigation and additional research.

Pirfenidone, another antifibrotic agent, has demonstrated efficacy in the treatment of idiopathic pulmonary fibrosis (IPF) (King et al., 2014) and has also been investigated in the context of unclassifiable progressively fibrosing ILD (Maher et al., 2020). While a study on the latter did not meet its primary endpoint, as defined by the change in forced vital capacity (FVC) measured by telemetry, a significant improvement in FVC measured on-site was observed. This suggests the potential efficacy of pirfenidone in this specific indication. In the PIRFS study, pirfenidone or placebo was introduced to patients with fibrotic sarcoidosis, but the results were inconclusive due to a low number of participants (Baughman et al., 2021b).

This narrative review aims to provide a comprehensive examination of the theoretical background and existing evidence surrounding the consideration of antifibrotic agents, such as pirfenidone and nintedanib, in the management of progressive fibrosing pulmonary sarcoidosis. By weighing the pros and cons, the review seeks to contribute to understanding whether antifibrotics should be incorporated into the therapeutic approach for progressive fibrosing pulmonary sarcoidosis.

## Pathogenesis of sarcoidosis

### Sarcoid antigens

Due to the activation of both the innate and adaptive immune responses, it is plausible that certain antigens play a pivotal role in the pathophysiology of granuloma formation and inflammation in sarcoidosis. These antigens can be broadly categorized into two groups: inorganic and organic. Inorganic antigens, such as silicates, dusts, and metal fumes, have been implicated in sarcoidosis pathogenesis. Notably, studies on construction workers with occupational exposure to silica reported a higher risk of sarcoidosis in two Swedish cohort studies (Vihlborg et al., 2017; Jonsson et al., 2019). Following the collapse of the World Trade Center (WTC) in 2001, individuals exposed to dust from destroyed construction and furnishing materials containing components like calcite, gypsum, bassanite, and silica, exhibited an increased incidence of sarcoidosis, particularly among NYC firefighters actively engaged during the WTC emergency response (McGee et al., 2003; Izbicki et al., 2007). Lymphocyte proliferation tests in metal dust and fumes exposure cases have demonstrated an elevated risk of sarcoidosis (Fireman et al., 2016; Beijer et al., 2020).

Among organic factors, the potential involvement of *Mycobacterium* tuberculosis and *Cutibacterium* acnes is noteworthy. The Kveim test, involving the intradermal injection of sarcoid lymphoid tissue, resulted in cutaneous granuloma formation after 4–6 weeks, suggesting a potential link to an infectious agent. Exposures to microbial heat shock proteins (HSPs), particularly mycobacterial HSPs with similarities to human HSPs, have been hypothesized to trigger a sarcoid-like immune response leading to granuloma formation via innate and adaptive immune cells and pattern recognition receptors (Eishi et al., 2002; Inaoka et al., 2019). Studies have reported higher concentrations of specific mycobacterial HSPs, such as Mtb-HSP70, Mtb-HSP65, and Mtb-HSP16 in sarcoidosis patients’ lymph nodes, sera, and immune complexes (Dubaniewicz, 2023). Additionally, *Cutibacterium* acnes has been identified in granulomas and inflammatory cells of lymph nodes, suggesting a potential role in the pathogenesis of sarcoidosis (Negi et al., 2012; Zhou et al., 2015; Suzuki et al., 2018).

### Early inflammation

The pathophysiological process of sarcoidosis primarily revolves around granuloma formation, predominantly affecting the lungs, lymph nodes, and other organs. Unlike infectious diseases like tuberculosis, sarcoidosis entails the development of noncaseating granulomas. Antigen-presenting cells (APCs), including macrophages, dendritic cells, and epithelial cells, present antigens via the MHC II-TCR complex to activated CD4^+^ T cells in genetically predisposed individuals. These CD4^+^ T cells comprise various subsets, including Th1, Th17, and Treg cells, which play pivotal roles in the inflammatory cascade. Th1 cells are crucial for granuloma formation and secrete cytokines such as IL-2, interferon-gamma (IFN-gamma) (Robinson et al., 1985; Pinkston et al., 1983) and CXCL10, CXCR3, IL-12R, IL-18R which help with APCs migration and activation (Katchar et al., 2003; Miotto et al., 2001; Szabo et al., 2000; Matsuda et al., 2007). Meanwhile, Th17 cells, a newer aspect in sarcoidosis pathophysiology, produce IL-17, contributing to the induction and maintenance of the disease process (Berge et al., 2012) by producing cytokines such as IL-17, IL-17F, IL-22, IL-26, IFN-gamma, and CCL20 (Boniface et al., 2008). Additionally, a special subset of Th17 cells, Th17.1, formed through IL-12 and IFN-gamma co-signaling (Duhen and Campbell, 2014; Zielinski et al., 2012), further exacerbates granuloma formation. The role of Th17.1 is crucial in granuloma formation–there is a higher level of Th17.1 in peripheral blood, BALF, and granuloma tissue (Broos et al., 2018; Richmond et al., 2013; Tøndell et al., 2014) in group of sarcoidosis patients. Regulatory T cells (Treg) provide immunosuppressive functions, inhibiting TNF-alpha and exerting antiproliferative effects on other Th cells (Miyara et al., 2006). While other immune cells like natural killer (NK) cells play minor roles, they also contribute to the inflammatory milieu with both pro- and anti-inflammatory substances, especially a particular type of NK cells - CD56 – is more frequent in BALF than in peripheral blood, which can also produce TNF-alpha and IFN-gamma (Katchar et al., 2005).

### Healing

The transition from early inflammation to healing in sarcoidosis involves a shift in the composition and activation of lymphocytes and macrophages. Macrophages, crucial in the inflammatory process, can be divided into two groups: M1 and M2. M1 macrophages exhibit proinflammatory activation, while M2 macrophages display anti-inflammatory and profibrotic properties (Italiani and Boraschi, 2014). In sarcoidosis, the balance between M1 and M2 polarization remains ambiguous, with mixed findings reported (Locke et al., 2019; Honda et al., 2016; Wikén et al., 2010; Prokop et al., 2011). During the acute and fibrotic phase of bleomycin-induced lung injury, the M2 macrophages are overexpressed (Misharin et al., 2013). Elevated M2 levels and significant transforming growth factor (TGF-beta) expression are observed in muscular sarcoidosis, contributing to granuloma formation and fibrosis development (Prokop et al., 2011). Additionally, the recruitment and differentiation of CD4^+^ T cell subgroups, particularly Th1, Th17, and Treg cells, influence the healing process. Th1 cells continue to play a role in granuloma formation during the healing phase, while Th17 cells and their subset Th17.1 contribute to sustained inflammation and granuloma development. Regulatory T cells modulate the inflammatory response, albeit incompletely inhibiting proinflammatory cytokines like TNF-alpha. Throughout this healing phase, the involvement of immune cells, such as natural killer cells, persists, contributing to the intricate balance between inflammation and resolution in sarcoidosis. We can divide patients into two groups, based on the cytokine profile–profibrotic, which mostly comprises M2 macrophages, Th2 and Treg lymphocytes and their cytokines such as IL-4, IL-5, IL-7, IL-10, IL-13, TGF-beta and CXCL18, and nonfibrotic, which consists of M1 macrophages, Th1 and Th17.1 lymphocytes and their cytokines such as IFN-gamma, TNF-alpha, IL-1Beta, IL-6, IL-13, IL-17 and CXCL9/10/11 (Asif et al., 2023; Zhang et al., 2021).

## Lung fibrosis in sarcoid patients

### Clinical picture

In a group of patients with fibrotic sarcoidosis respiratory symptoms are most common, such as cough and dyspnea. In contrast with the majority of fibrotic ILD wheezing occurs more frequently due to bronchial distortion and central airway bronchiectasis. It may also relate to higher risk of bacterial infections, sarcoidosis exacerbation and even hemoptysis (Judson, 2017). Hemoptysis may also be associated with mycetoma’s consisting of Aspergillus fungi masses (Pena et al., 2011). Moreover, hemoptysis and signs of hypoxemia, might be symptoms of sarcoidosis associated pulmonary hypertension (SAPH). The incidence of SAPH is higher in radiological stage IV and varies between 5% and 20% of patients with sarcoidosis (Baughman et al., 2015). The mechanism of SAPH is multifactorial, mostly comprise of fibrosis of interstitial space, formation of granulomas nearby of vessels, which may result in vascular obstruction, and granulomatous vascular inflammation (Bandyopadhyay and Humbert, 2020). SAPH is the most crucial predictor of mortality in fibrotic sarcoidosis and may be connected even with an eight-fold higher risk of mortality, resulting in a median survival of 5.7 years (Tiosano et al., 2019; Nardi et al., 2011). Risk factors of SAPH are severe dyspnea, hypoxia, 6-minute walking distance less than 300 m and forced vital capacity (FVC)/transfer capacity for carbon monoxide (TLCO) over 1.5. Chronic fatigue syndrome frequently occurs during sarcoidosis and might be interpret by some clinicians as a symptom or manifestation of depression (Górski and Piotrowski, 2016). Severe or fibrotic sarcoidosis, like other fibrotic ILDs, frequently correlates with depression, underscoring the importance of a comprehensive approach to managing comorbidities like depression (Tzouvelekis et al., 2020; Borson and Randall Curtis, 2001).

**FIGURE 1:**
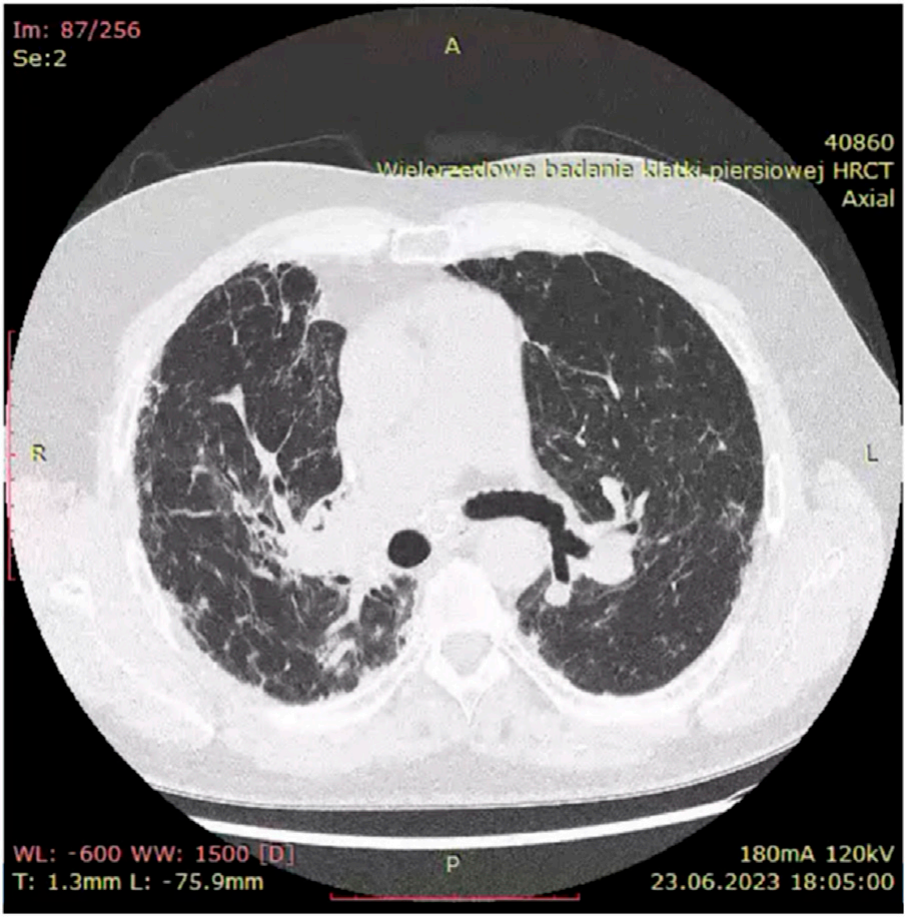
Bronchial distortion pattern in fibrotic sarcoidosis with bronchial angulation visible especially on right upper bronchi.

**FIGURE 2:**
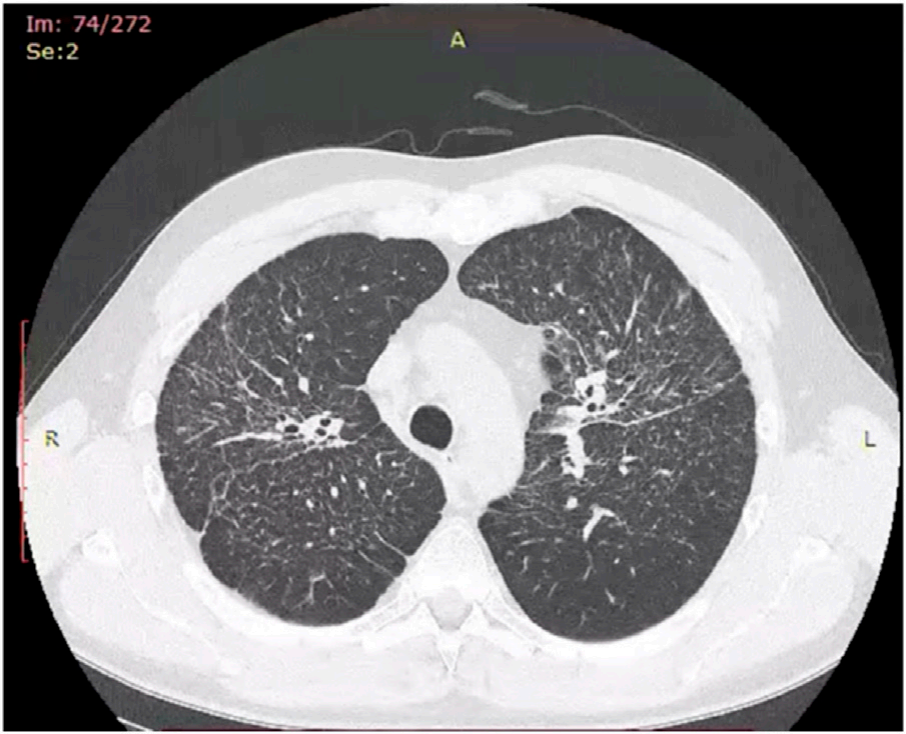
Linear scarring pattern in fibrotic sarcoidosis in right middle lobe.

**FIGURE 3:**
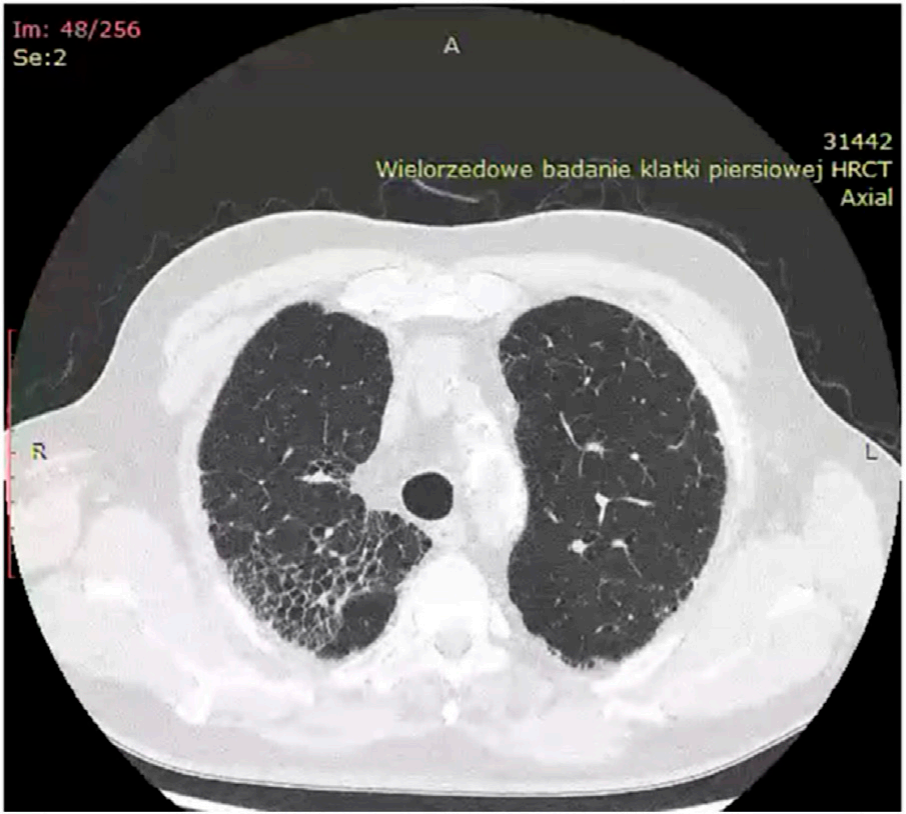
“Honeycombing” pattern in fibrotic sarcoidosis–located in right upper lobe.

Historically, X-ray examination was used as diagnostic tool for fibrotic sarcoidosis (stage IV sarcoidosis). Today, high resolution tomography (HRCT) is the best tool for diagnosis of fibrotic sarcoidosis. Three HRCT patterns: bronchial distortion, linear scarring, and honeycombing have been distinguished (Sverzellati et al., 2010). Bronchial distortion (Figure 1), which usually originates from massive lymphadenopathy and consolidations of inflammatory infiltrations nearby bronchi, is manifested by bronchial angulation and bronchiectasis. Linear scarring (Figure 2), which usually originates from broncho-vascular bundles involvement, is mostly located in lower lobes. Honeycombing (Figure 3), which in fibrotic sarcoidosis is improperly called “UIP-like pattern”, consists of cysts, which are bigger than in UIP pattern. It is mostly located in upper lobes and originates from ground-glass opacities involvement. Moreover, UIP pattern is present in other ILDs such as connective-tissue disease-interstitial lung disease (CTD ILD). Due to this fact, some scientists differentiate UIP pattern in IPF and in CTD-ILDs with new, three radiological patterns – “straight edge” sign - isolation of fibrosis to the lung bases without substantial extension along the lateral margins of the lungs on coronal images, “anterior upper lobe” sign - concentration of fibrosis within the anterior parts of the upper lobes, and “exuberant honeycombing” sign - widespread formation of honeycomb-like cysts in more than 70% of the fibrotic areas of the lungs. These three signs were significantly more common in patients with CTD-ILD UIP-pattern rather than typical IPF UIP pattern (Chung et al., 2018). In case of UIP-like pattern in sarcoidosis fibrosis there were no articles about this three radiological signs, but “exuberant honeycombing” sign seems to be more frequent in our single-center observation and these signs need further evaluation in other fibrotic diseases, such as sarcoidosis.

When it comes to pulmonary function test results (PFTs), restrictive pattern is common, with TLC (total lung capacity), VC (vital capacity) and TLCO reduction (Spagnolo et al., 2018). In contrast to other ILDs obstructive disease is also frequent, mostly due to bronchial distortion and bronchiectasis. In most fibrotic sarcoidosis cases PFTs results are stable during whole process. In six-minute walk tests, people with fibrotic sarcoidosis typically walk shorter distances (Baughman et al., 2007). This reduced walking ability is linked to several serious health issues, including pulmonary hypertension associated with sarcoidosis, decreased FVC, and low oxygen levels during exercise (Gupta et al., 2022).

Lung transplants for sarcoidosis patients constitute 2%–5% of all lung transplant cases (Spagnolo et al., 2018). There are no available data about which sarcoidosis patients are qualified, but probably Scadding’s stage IV patients may overrepresent this small group. In one study, mPAP of > = 30 mmHg in a group of sarcoidosis patients is an independent risk factor of mortality on the waiting list and this group has higher waitlist mortality than COPD patients. Patients with sarcoidosis with mPAP less than 30 mmHg has the lowest waitlist mortality comparing to COPD and IPF (Sosa et al., 2023). Patients with fibrotic sarcoidosis with lung involvement over 20% have higher mortality than other patients (Jeny et al., 2020).

### Risk factors

Fibrotic sarcoidosis might be seen in up to 20% of patients with sarcoidosis (Judson et al., 2012; Moller, 2003). The average age of presentation with fibrotic pulmonary sarcoidosis is in the fourth decade of life (Nardi et al., 2011). It is not clear if race is a risk factor of fibrotic sarcoidosis. In one study African American patients were less likely to present stage I disease, but rate of stage IV was similar between African American and Caucasian (Rybicki et al., 1997). In another study black race and females have significantly higher risk of higher radiological Scadding grade (Judson et al., 2012).

Genetic risk factors of fibrotic sarcoidosis are focused on single nucleotide polymorphisms (SNPs). Variations in certain genes, specifically those encoding gremlin for tissue repair (GREM1) (Heron et al., 2011), caspase recruitment domain-containing protein 15 (CARD15) - also referred to as nucleotide-binding oligomerization domain containing protein 2 (NOD2) (Sato et al., 2010), and the cytokine transforming growth factor (TGF) β3 (Kruit et al., 2006), increase susceptibility to fibrosis in sarcoidosis. Also, one study on Caucasian patients revealed that a promoter polymorphism in prostaglandin-endoperoxide synthetase 2 (PTGS2) was linked to a higher risk of SAPF (Hill et al., 2006). Sarcoidosis associated pulmonary fibrosis has some unique features such as variants of annexin A11 and PVT1. Annexin A11 (ANXA11) is a calcium-dependent membrane-binding protein that has been linked to the risk of developing fibrotic sarcoidosis (Mirsaeidi et al., 2016a). In a small study involving African - American patients with sarcoidosis, certain ANXA11 single nucleotide polymorphisms (SNPs), namely rs1049550 and rs12779955, were associated with an increased susceptibility to pulmonary fibrosis. Those carrying the T genotype of rs1049550 had a 4.5 times higher risk of developing pulmonary fibrosis (Mirsaeidi et al., 2016b). Also, SNPs variants of PVT1 (plasmacytoma variant translocation 1) gene were associated with increased susceptibility to fibrosis in group of patients of African American descent with sarcoidosis (Garman et al., 2024). Telomere’s length, and especially terminal restriction fragment (TRF), are potentially new factor of fibrosis, like in other ILDs, such as IPF (Prokop et al., 2011; Bilgili et al., 2019). A significant decrease of TRF was observed in sarcoidosis comparing to control group (Saito et al., 2018). In one study no significant difference was shown in TRF length between stage I, II, and III, but stage IV was not included (Ma and Meng, 2019). A specific genetic variation, −765G>C, in the prostaglandin-endoperoxide synthase 2 (PTGS2) gene has been linked to an increased risk of fibrotic sarcoidosis (Hill et al., 2006). PTGS2 is an enzyme that plays a crucial role in producing prostaglandin E2, a substance known for its antifibrotic activity. People with the −765C variant of this gene are more susceptible to sarcoidosis, tend to have a worse prognosis, and are more likely to develop fibrotic disease.

From occupational and environmental perspective of fibrotic sarcoidosis silica exposure might relate to fibrotic process in patients with sarcoidosis (Beijer et al., 2021a). Apart from that, there is lack of evidence of any other factors that might play a role in the pathogenesis of fibrotic sarcoidosis.

### Pathogenesis

Fibrosis of the lungs in individuals with sarcoidosis typically commences following the formation of granulomas. The gene expression profiles of patients with progressive sarcoidosis, many of whom exhibited fibrosis on imaging, closely resembled those with inflammatory hypersensitivity pneumonitis (Lockstone et al., 2010). Initially, Th1 lymphocytes, in conjunction with M1 macrophages, contribute to granuloma formation. However, there is a transition towards Th2 lymphocytes and M2 macrophages, both of which exhibit pro-fibrotic properties (Moller, 1999; Teirstein and Morgenthau, 2009). Elevated levels of Th2 cytokines such as IL-4, IL-5, IL-9, IL-10, IL-13, and TGF-beta have been linked to increased extracellular matrix production. IL-13 can boost TGF-beta production while reducing TNF-alpha release (Kunkel et al., 1996). Studies have indicated higher levels of IL-13, IL-5, and IL-7 in individuals with pulmonary fibrosis (Patterson et al., 2013; Hauber et al., 2003). IL-4, CCL-2 and IL-13 encourage the proliferation of M2 macrophages, which in turn stimulate fibroblast activity through the release of TGF-beta and other molecules (Locke et al., 2019; Liu et al., 2011; Shamaei et al., 2018). The upregulation of CCL-8 and CCL-18 during the Th2/M2 shift may also contribute to pulmonary fibrosis and collagen production (Luzina et al., 2006; Prasse et al., 2006). M2 macrophages produce arginase via the expression of the Arg1 gene, which converts arginase into ornithine, a precursor of collagen (Munder et al., 1998). Additionally, M2 activation has been observed in the fibrosis of neuromuscular sarcoidosis through the overexpression of CD206, CD301, and Arg-1 (Prokop et al., 2011). Moreover, patients with fibrotic radiographic stages of sarcoidosis have higher proportion of Tregs, and lower proportion of Th17.1 (Zhang et al., 2023). Also, platelet-derived growth factor (PDGF), which is secreted by Tregs, regulates the proliferation of lung fibroblasts and collagen deposition (Matsuse, 1998).

TGF-beta, potentially secreted by Th2 lymphocytes and M2 macrophages, likely plays a pivotal role in lung fibrosis. Overexpression of TGF-beta/Smad signaling has been observed in various extrapulmonary fibrotic conditions (Saito et al., 2018; Ma and Meng, 2019; Piotrowski et al., 2015a; Xu et al., 2016). TGF-beta1 is primarily responsible for collagen deposition, fibroblast recruitment, and myofibroblast transformation from epithelial cells (Gharaee-Kermani et al., 2009; Coker et al., 2001; Goodwin and Jenkins, 2009), while parenchymal involvement has been linked to TGF-beta3 (Piotrowski et al., 2014). Moreover, the frequencies of TGF-beta3 single nucleotide polymorphisms (SNPs) varied between individuals with fibrotic sarcoidosis and chronic sarcoidosis, where chronic sarcoidosis was characterized by the persistence of symptoms for a minimum of 2 years or experiencing two or more disease flares (Pabst et al., 2011). On the other hand, TGF-beta1 gene polymorphisms were not associated with fibrosis (Kruit et al., 2006). Bone morphogenic proteins (BMPs), which are like TGF-beta, stimulate tissue regeneration, but they are not profibrotic comparing to TGF-beta. The balance between BMPs and TGF-beta may play an important role in fibrotic process. Inhibitors of BMPs, such as gremlin, were associated with pulmonary sarcoidosis with fibrosis (Heron et al., 2011).

The Wnt signaling pathway, particularly Wnt-beta, has been identified in fibrotic foci of idiopathic pulmonary fibrosis (IPF) (Bartczak et al., 2020; Königshoff et al., 2008; Chilosi et al., 2003). In pulmonary sarcoidosis, there is an upregulation of Wnt5A, Wnt7A, Wnt7B, and B-catenin signaling in bronchoalveolar lavage fluid cells (Levänen et al., 2011). Furthermore, there is a correlation between B-catenin signaling and epithelial-mesenchymal transition, which sustains fibrosis (Huang et al., 2021).

Recent studies have conducted direct comparisons between fibrotic sarcoidosis and other fibrotic interstitial lung diseases (ILDs), yielding insightful findings. These investigations have primarily focused on the repair and plasticity of alveolar epithelial cells. MRP14 (myeloid-related protein 14), also known as S100A9, is intracellular calcium-binding protein, was found to be significantly elevated in bronchoalveolar lavage (BAL) samples from both sarcoidosis and idiopathic pulmonary fibrosis (IPF) patients, and its levels correlated with the stage of chest radiographs in sarcoidosis (Korthagen et al., 2010). One study found the impact of S100A9 on human embryo lung fibroblasts, revealing its role in promoting fibroblast proliferation and the deposition of type III collagen (Lewis and Kirkwood, 1990). Matrix metalloproteinases (MMPs) play a crucial role in modifying the lung microarchitecture through processes such as fibroblast expansion, myofibroblast differentiation, and accumulation of extracellular matrix. However, contrary to the observed elevation of MRP14/S100A9, serum levels of MMP1 and MMP7 were notably higher in IPF compared to fibrotic sarcoidosis (Morais et al., 2015). These differing MMP signatures may have implications for lung remodeling, especially concerning the development of usual interstitial pneumonia (UIP) in IPF as opposed to other patterns of fibrotic changes observed in non-IPF ILDs, like sarcoidosis. Moreover, ILDs with fibrotic features share imbalance in phosphatase/kinase activation which results in extracellular matrix deposition. For example, Mitogen-activated protein kinase (MAPK) phosphatase 5 (MKP-5) negatively regulates p38 MAPK. In one study MKP-5-deficient mice were protected from the development of lung fibrosis, expressed reduced levels of hydroxyproline and fibrogenic genes, and displayed marked polarization towards an M1-macrophage phenotype (Xylourgidis et al., 2019). Moreover, profibrogenic effects of the transforming growth factor-β1 (TGF-β1) were inhibited in MKP-5-deficient lung fibroblasts. MKP-5-deficient fibroblasts exhibited enhanced p38 MAPK activity, impaired Smad3 phosphorylation, increased Smad7 levels, and decreased expression of fibrogenic genes. Polarization towards M1 macrophages and their activation may be influential on pathogenesis of fibrotic sarcoidosis. Also, enhanced p38 signaling and bigger production of TNF-α and IL-12/IL-23p40 on stimulation with NOD1 and TLR4 agonists in response to microbial products is caused by abnormal regulation of MAPK phosphatase and contributes to heightened inflammation in sarcoidosis (Rastogi et al., 2011). A recent analysis of lung tissue from sarcoidosis patients undergoing transplantation showed that while most had fibrosis related to granulomatous inflammation, a minority exhibited alternative mechanisms of lung fibrosis. This included cases where granulomas were replaced by scar tissue or where granulomatous inflammation ceased while other lung diseases progressed. Some patients were misdiagnosed with sarcoidosis when they had granulomatous lymphadenopathy alongside another lung disease (Goodwin and Jenkins, 2009).

Different types of Toll-like receptors (TLRs) might also have a significant role in fibrotic sarcoidosis. TLRs have the potential to trigger the innate immune system, enabling it to recognize and regulate interactions between the innate system and other antigens (Fitzgerald and Kagan, 2020). In chronic sarcoidosis, TLR2 polymorphisms appear to be more common (Veltkamp et al., 2007). Additionally, the haplotype of single nucleotide polymorphism (SNP) variants that affect TLR1, TLR6, and TLR10 genes—acting as co-receptors with TLR2—was found to be absent in the fibrotic group (Veltkamp et al., 2012). The TLR3 polymorphism Leu412Phe (rs3775291), previously linked to accelerated disease progression and increased mortality risk in idiopathic pulmonary fibrosis (IPF) (O’Dwyer et al., 2013), was examined in sarcoidosis patients and correlated with the progression of the disease to fibrosis (Cooke et al., 2018), in the mechanism of reduced apoptosis of fibroblasts and increased production of CCL-18, which also occurs in IPF. Serum amyloid antigen (SAA) is another factor that is elevated in fibrotic pulmonary diseases, including sarcoidosis (Beijer et al., 2021b; Chen et al., 2010). Another aspect is dysregulation of the mammalian target of rapamycin (mTOR). In cases of fibrotic pulmonary sarcoidosis mTOR complex 1 (mTORC1) is upregulated, which results in excess granuloma formation (Vukmirovic et al., 2021). Recent studies have shown that the hypoxia-induced factor 1-alpha (HIF1α) pathway is important in granulomatous diseases (Piotrowski et al., 2015b). When monocyte-derived macrophages are under low oxygen conditions, they increase their inflammatory responses but are less effective at presenting antigens to T-cells, which decreases the immune response. These macrophages also release a substance called plasminogen activator inhibitor-1 (PAI-1). PAI-1 can help granulomas to form, and it can also contribute to the development of fibrotic disease (Jeny et al., 2021).

### Antifibrotics; mechanisms of action, possible points of interaction with sarcoidosis pathophysiology

Pirfenidone (5-methyl-1-phenyl-2-[1H]-pyridone) was first known as an anti-inflammatory and antioxidant agent (Iyer et al., 1999a). Its antifibrotic properties were first shown in experimental models of lung fibrosis, where it suppressed elevation of lung basic-fibroblast growth factor (bFGF) and transforming growth factor (TGF)-beta1 levels (Oku et al., 2008). In further studies it decreased the expression of TGF-beta gene at the transcriptional level (Iyer et al., 1999b) and significantly downregulated the bleomycin-induced overexpression of procollagen genes (Iyer et al., 1999a). Pirfenidone attenuated lung fibrosis in various animal models (Schaefer et al., 2011). It was shown to reduce hydroxyproline accumulation in the lung, both when treatment was concurrent with bleomycin administration and when used after the instillation of bleomycin (Oku et al., 2008). Pirfenidone also reduced the activity of prolyl hydroxylase, a marker of collagen synthesis, and the collagen mRNA expression in hamsters’ lungs subjected to single bleomycin administration (Iyer et al., 1999a).

Many studies showed that pirfenidone can reduce the TGF-beta expression effectively (Schaefer et al., 2011). The drug alleviates pulmonary fibrosis by regulating Wnt/GSK-3β/β-catenin and TGF-β1/Smad2/3 signaling pathways (Lv et al., 2020). Other important mediators that may be important in the pathogenesis of lung fibrosis and influenced by pirfenidone include metalloproteinases and growth factors other than TGF-beta. Effects on growth factors include the downregulation of PDGF and FGF. In models of cardiac and liver fibrosis pirfenidone normalized expression of MMP-2 and MMP-9 (Di Sario et al., 2004; Lee et al., 2006).

Its anti-inflammatory properties, as shown in *in vitro* models are multiple. It alleviated the bleomycin-induced production of IL-1beta, IL-6, IL-12p40, and monocyte chemoattractant protein (MCP)-1, stroma cell-derived factor (SCDF, CXCL12), and IL-18 (Oku et al., 2008). CXCL12 is responsible for fibrocyte trafficking to the lung (Phillips et al., 2004).

Nintedanib is a small-molecule tyrosine kinase inhibitor, that targets receptors of several growth factors, namely fibroblast growth factor (FGF, receptors 1–3), platelet-derived growth factor (PDGF, receptors alpha and beta), and vascular-endothelial growth factor (VEGF, receptors 1–3). By inhibiting the above-mentioned receptors, it exerts an indirect inhibitory effect on the main pro-fibrotic cytokine, transforming growth factor beta (TGF-beta). In addition, it inhibits the Src family kinase lymphocyte-specific tyrosine protein kinase (Lck), colony-stimulating factor (CSF)-1 receptor (CSF1R) and many other kinases (Wollin et al., 2019).

Nintedanib exerts direct antifibrotic activity by attenuating the influx of fibrocytes from the blood to the lung and their differentiation to fibroblasts (Sato et al., 2017). Nintedanib also inhibits the motility of fibroblasts, as it was proven on cells from IPF patients, and inhibits the PDGF-induced contraction of human lung fibroblasts on collagen gels (Wollin et al., 2019). It inhibits fibroblast to myofibroblast transmission induced by TGF-beta (Wollin et al., 2014), and release of collagen from fibroblasts after stimulation with TGF-beta (Wollin et al., 2019).

Nintedanib exerts anti-inflammatory activity *in vitro*, which may be particularly important in sarcoidosis. It may interfere with many molecular pathways involved in the pathogenesis of sarcoid granulomatous inflammation. Lck is a 56 kD lymphocyte-specific kinase, a member of the Src kinase family. It is responsible for early propagation and modulation of T-cell receptor (TCR). It is required for T-cell proliferation and production of interleukin-2 (IL-2). Nintedanib was shown to inhibit Lck at IC50 comparable or even lower than that of VEGF-R1,2,3 inhibition (Hilberg et al., 2008). It inhibits a few cytokines of Th1-type of inflammation, such as IL-2, IL-12p70 and interferon-gamma (Wollin et al., 2019). In this way, it may influence the early stages of inflammation, shortly after the contact of the sarcoid antigen with immune cells. Nintedanib was also shown to inhibit many Th2-type of inflammation-related cytokines, such as IL-4, IL-5, IL-10, and IL-13 (Wollin et al., 2019). It also inhibits polarization of macrophages, preventing transformation of M1 to M2. A marker of macrophage polarization is a profibrotic chemokine, CCL18. The decrease in CCL18 production may be related to an inhibitory activity of nintedanib exerted on CSF1R. Nintedanib prevented the CSF1-induced phosphorylation of CSF1R and activation of the downstream signalling pathways, thus preventing the transformation of macrophages to profibrotic M2a phenotype (Bellamri et al., 2019). In animal models, Nintedanib attenuates the accumulation of lymphocytes and IL-1beta in bronchoalveolar lavage fluid and the proliferation of alveolar macrophages in the bleomycin lung fibrosis model in a mouse (Ackermann et al., 2017).

### Antifibrotics in sarcoidosis – to whom?

Antifibrotics in case of fibrotic sarcoidosis might be helpful by its influence on the course of progresive pulmonary fibrosis. Progresive pulmonary fibrosis (PPF) is a term characterized by 3 aspects: worsening respiratory symptoms, physiological evidence of respiratory progression (absolute decline in FVC ≥5% predicted within 1 year or absolute decline in TLCO (corrected for Hb) ≥10% predicted within 1 year) and radiological evidence of disease progression (increased extent or severity of traction bronchiectasis and bronchiolectasis, new ground-glass opacity with traction bronchiectasis, new fine reticulation, increased extent or increased coarseness of reticular abnormality, new or increased honeycombing, increased lobar volume loss) (Raghu et al., 2022). From our point of view, worsening of PTFs results seems to be less frequent in group of fibrotic sarcoidosis comparing to other fibrotic ILDs. In fibrotic sarcoidosis criteria of radiological progression play key role in PPF characteristics. In ILDs other than IPF, such as fibrotic sarcoidosis, the progression pattern is variable and may include the evolution of ground-glass abnormalities to reticular abnormalities, reticular abnormalities to honeycombing, and/or an increase in traction bronchiectasis/bronchiolectasis. For example, the presence of honeycombing and traction bronchiectasis, associated with worse prognosis, along with a greater extent of fibrotic changes, is predictive of mortality in IPF, rheumatoid arthritis-related ILD, systemic sclerosis-related ILD, fibrotic HP, and fibrotic sarcoidosis (Walsh et al., 2014). For treatment of PPF there were two potential antifibrotic substances, which were mentioned before – pirfenidone and nintedanib. Pirfenidone, which was evaluated in RELIEF trial, showed a slower decline in percent predicted FVC, but the study was terminated prematurely due to challenges related to slow recruitment in non-IPF progressive fibrotic lung disease (Behr et al., 2021). Moreover, the study excluded patients with sarcoidosis, limiting its applicability to this cohort. Whereas nintedanib, which efficacy was evaluated during INBUILD trial, proved its efficacy on lowering disease progression measured by FVC decline both in patients with PPF UIP-pattern and PPF non-UIP pattern (fibrotic sarcoidosis included in this group) (Flaherty et al., 2019). In case of fibrotic sarcoidosis, the limitation of this study was a small number of sarcoidosis patients included in the study – 12. In this case, there is need for new trials for assessment of nintedanib in fibrotic sarcoidosis with bigger number of participants. Efficacy of nintedanib was proved in other fibrotic ILDs, such as interstitial lung disease associated with systemic sclerosis (Distler et al., 2019), and fibrotic hypersensitivity pneumonitis (f-HP) (Tzilas et al., 2020) – in this case sarcoidosis shares many characteristics with HP such as granuloma formation. Although high-quality studies specifically focusing on populations with sarcoidosis-related fibrosis are lacking, initiation of antifibrotic therapy is suggested based on guidelines for manifestations of progressive fibrotic phenotype (PPF). The use of antifibrotics, such as nintedanib, may be justified to slow disease progression and improve lung function in patients with pulmonary fibrosis, including those with sarcoidosis.

**FIGURE 4:**
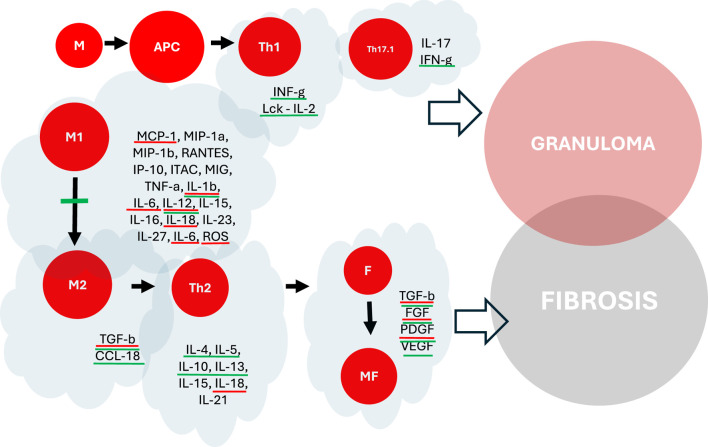
The impact of pirfenidone and nintedanib on the pathogenesis of granuloma formation and fibrosis in pulmonary sarcoidosis. Molecules highlighted in red - potential interaction with pirfenidone. Molecules highlighted in green - potential interaction with nintedanib. M, Macrophages (M1, M2), APC, Antigen Presenting Cells, T, T-Lymphocytes (Th1, Th17.1, Th2), F, Fibroblasts, MF, Myofibroblasts.

Antifibrotics may interfere with many aspects and mechanisms of sarcoidosis pathogenesis, from early stages of inflammation up to late stages, leading to irreversible scarring (Figure 4). The anti-inflammatory properties of pirfenidone and nintedanib could help treat sarcoid patients in the early stages of the disease. However, this concept is very controversial and highly hypothetical. Clinical studies should be conducted to compare their effectiveness with all recommended anti-inflammatory drugs, such as steroids, conventional immunosuppressants, and anti-TNF agents. However, if we consider fibrosis as a continuum and consequence of chronic and self-perpetuating granulomatous inflammation, such an intervention would not be senseless if antifibrotics were introduced in selected patients with the highest risk of developing fibrosis.

In patients in whom fibrosis is an end-stage of the long-lasting disease, and when the activity of inflammation is dubious or negligible, such a treatment would be, without doubt, pointless. PET examination would be highly desirable to answer this dilemma. Therefore, the question is whether the fibrotic process in sarcoidosis may be, at least in some cases, progressive and independent of preceding inflammation. In these instances, fibrosis would be a self-perpetuating process like IPF.

Based on the results of chest CT scans and PET examination, fibrotic sarcoidosis could be divided into active progressive pulmonary fibrosis and inactive, “burnt-out” disease (Bandyopadhyay and Mirsaeidi, 2023). This intelligent and reasonable approach would allow for an objective selection. Lack of the effects of all three anti-inflammatory treatment grades should be proven in the 3–6 months trial. The number of patients who meet these criteria will probably be minimal.

## Conclusion

Sarcoidosis, which is the most common interstitial lung disease, is still a mystery for patients, clinicians, and scientists. Its pathogenesis and multiorgan involvement question current diagnostic and therapeutic approaches. Extracellular matrix collagen, which starts during granuloma formation, states that fibrosis is an integral part of the pathophysiology of sarcoidosis. In a pathophysiological way, sarcoidosis shares many characteristics with IPF and other fibrotic diseases, such as similar cytokines secreted by fibroblasts and other cells. On the other hand, primary granuloma formation and its persistence, which constitutes sarcoidosis, varies from other ILDs such as IPF. Pulmonary complications, poor prognosis, and lack of effective treatment prompt new therapeutic approaches. Nintedanib, which inhibits many profibrotic factors, is proven to be an effective medication for patients with progressive pulmonary fibrosis. Its other properties, such as influence on granuloma formation, inflammation inhibition and interference with subtypes of macrophages, are promising for effective antifibrotic therapy in fibrotic sarcoidosis. However, it is still unknown whether antifibrotic therapies might be helpful. More randomized controlled trials (RCTs) still need to focus on antifibrotics in fibrotic sarcoidosis. Without this, sarcoidosis will remain a neglected disease.
